# Risk-Stratified and Response-Adapted Therapy for Pediatric Hodgkin Lymphoma in Argentina: The GATLA Experience

**DOI:** 10.1155/ah/5453729

**Published:** 2025-07-26

**Authors:** David Veron, Patricia Streitenberger, Mónica Matus, Pedro Negri Aranguren, Alejandra Costa, Daniela Morell, Sergio Terrasa, E. Mauricio Castellanos, Pedro de Alarcon, Eduardo Dibar, Mónica Makiya

**Affiliations:** ^1^Hospital Universitario Austral, Pilar, Buenos Aires, Argentina; ^2^Hospital Italiano de Buenos Aires, Buenos Aires, Argentina; ^3^Grupo Argentino Para el Tratamiento de Leucemias Agudas GATLA, Buenos Aires, Argentina; ^4^Hospital Materno Infantil San Roque, Paraná, Entre Ríos, Argentina; ^5^Hospital de Niños Sor María Ludovica, La Plata, Argentina; ^6^Hospital Infantil de Córdoba, Córdoba, Argentina; ^7^Departamento de Investigaciones, Hospital Italiano de Buenos Aires, Buenos Aires, Argentina; ^8^AHOPCA, Unidad Nacional Oncología Pediátrica, Guatemala City, Guatemala; ^9^Department of Pediatrics, University of Illinois College of Medicine, Peoria, Illinois, USA

**Keywords:** COPDAC, GATLA, Hodgkin, OEPA, pediatric

## Abstract

**Background:** The international cooperation between GATLA and AHOPCA with the support of St. Jude led to the adoption of the OEPA/COPDAC as a strategy to improve outcomes in high risk (HR) patients with HL. This study also includes the ABVD regimen for intermediate risk (IR) and low risk (LR) patients.

**Methods:** Patients were stratified by predefined risk assignment. HR was defined as a disease in stages II B, III B, and IV. Modality treatment: LR: ABVD × 4 ± IFRT (20 Gy); IR: ABVD × 6 ± IFRT (20 Gy); and HR: OEPA–COPDAC + IFRT (20/25 Gy). The staging and response were reviewed in a periodic discussion of presentation of cases in the group. Eligibility for radiotherapy: LR patients in partial response (PR) after 4 ABVD and IR patients in PR after 2 ABVD received IFRT. All HR patients received IFRT at 20 (complete response (CR)) or 25 Gy (PR) depending on the response achieved after the first two OEPA cycles.

**Results:** From November 2012 to June 2022, 203 pediatric patients were enrolled. A total of 171 patients were eligible in this analysis. HR: 98 patients (57.3%), IR: 52 patients (30.4%), and LR: 21 patients (12.3%). More than half of the patients were in stages III and IV and more than half also presented B symptoms. The response evaluation was performed by PET/CT in 147/171 patients (86%). A total of 68/171 patients (40%) did not received radiotherapy. Radiotherapy was omitted in 95% of the LR patients and 70% of the IR patients. The 10-year OS was 95% (90.7–97.6) for the 171 patients and 93% (85.3–96.4) for HR patients. The 10-year EFS was 91% (85.2–94.2) for the 171 patients and 87.8% (79.5–92.9) for HR patients.

**Conclusion:** The international cooperation made it possible to significantly improve the outcomes of patients with advance disease in Argentina compared with our previous experience (7-PHD-96: COPP–ABV × 6 + IFRT Bulky Disease or PR (20/25 Gy): 5yOS: 85%, 5yEFS: 67%), reduce the number of patients who required radiotherapy, and reproduce the European experience for HR patients in a totally different context.

## 1. Introduction

Cure rates for pediatric classical Hodgkin lymphoma (cHL) have dramatically improved over the last 40 years in high income countries (HICs), achieving event-free survival (EFS) and overall survival (OS) rates over 90% [1]. In low- and middle-income countries (LMICs), however, this is not always the case [2].

Survival in LMIC is affected by multiple factors including delay in diagnosis; poor health associated to malnutrition and concomitant infections; lack of resources for adequate pathologic diagnosis and proper staging; drug shortages and inadequate access to radiotherapy leading to delays in therapy; and socioeconomic hardship and cultural barriers leading to abandonment of therapy [3–7].

Since 2009, the Association of Pediatric Hemato-Oncology of Central America (AHOPCA) was able to improve survival in high-risk (HR) patients treated according to treatment group 3 (TG-3) of the GPOH-HD2002 protocol in a LMIC setting [8] (Supporting Figure 1).

The greater interaction between different groups facilitated by the International Symposium on Hodgkin Lymphoma in Children, Adolescents, and Young Adults (ISCAYAHL) starting in 2011 allowed GATLA and AHOPCA to adopt the same treatment strategy since 2012, seeking to reproduce the results of the GPOH study in Latin America. Through https://www.cure4kids.org, and later WebEx, pediatric oncologists from many Latin American countries and St Jude Children's Research Hospital met weekly to discuss the cases [9, 10].

In Argentina, since its creation in 1967, the GATLA group registered 1110 pediatric patients in 7 consecutive protocols for HL until 2012 (Supporting Figure 2).

The 7-P-HD-96 experience enrolled 351 patients younger than 16 years from 1996 through 2004. Patients with HR HL received COPP–ABV (doxorubicin, bleomycin, and vinblastine) × 6 + 20-Gy IFRT or 30 Gy to sites of bulky disease and partial response (PR), leading to a 5-year EFS of 67% and a 5-year OS of 85% [11, 12].

In 2010, the EHP-10 protocol prescribed 6 cycles of ABVD + 30 Gy IFRT for HR patients. The recruitment of patients was slow due to the investigators' opposition to treating patients with a strategy that invloves a reported relapse and progression rate of 30%. Therefore, EHP-10 closed early after only 29 patients were enrolled over 3 years, of which only nine had HR disease and of which three relapsed within the first year [9].

The adoption of the OEPA/COPDAC strategy for HR patients in the 11-EHP-12 protocol had the objective of improving EFS and OS in patients with HR disease [13, 14].

## 2. Methods

### 2.1. Patients

The study was conducted at 20 GATLA institutions from November 2012 through June 2022 after approval by local ethics committees. It was a prospective nonrandomized study on the epidemiology, stage, early response evaluation, and treatment outcome of children with HL. The GATLA member centers obtained written informed consent from patient and/or guardian according to institutional guidelines.

Eligible patients were younger than 19 years of age with biopsy-proven HL (lymphocyte predominant HL was not excluded) and had to be HIV negative. The histopathological analysis was performed at each center though centralized review was offered in cases of doubt.

The inclusion criteria also included echocardiogram with shortening fraction greater than 28%; adequate renal and liver function unless explained by HL infiltration; absence of pregnancy (beta-HCG negative) in female patients of reproductive age; adequate lung function with normal spirometry; and clinical and imaging characterization of all sites compromised by the disease.

Exclusion criteria were psychiatric disorders that prevented understanding of the scope of the treatment by the patient or the legal guardians, impossibility of performing proper staging by imaging, and serious concurrent illness.

### 2.2. Staging, Risk Assignment, and Treatment

The study was designed to treat patients according to three risk groups with chemotherapy alone, or chemotherapy plus radiotherapy depending on the patient's disease burden at diagnosis and early response to therapy.

High-complexity centers participating in the study offered treating HR patients referred from lower-complexity centers.

Patients were staged according to the modified Ann Arbor classification [15]. “B” symptoms were defined as recurrent unexplained fever (≥ 38.3°C), unintentional weight loss (≥ 10% body weight), or recurrent drenching night sweats. Bulky mediastinal mass was defined by the ratio of the diameter of the mediastinal mass divided by the transthoracic diameter at the dome of the diaphragm (M/T) greater than 0.33 on the anteroposterior (AP) chest radiograph. Bulky peripheral nodal disease was defined as a mass greater than 6 cm in its longest transverse diameter. Staging imaging included an AP chest x-ray and a corporal positron emission tomography (PET)/computerized tomography (CT) and/or contrast enhanced CT of the neck, chest, abdomen, and pelvis. Bone marrow biopsy was performed in patients with stage IIB, III and IV when PET/CT was not available.

Patients were assigned to the following three risk groups:

  Group 1 or low risk (LR): patients with stages IA, IIA supradiaphragmatic disease without or nonbulky mediastinal involvement and with < 4 nonbulky nodal sites of disease; or patients with stage IA, IIA infradiaphragmatic disease with < 4 nonbulky nodal sites of disease. These patients were treated with 4 cycles of ABVD (doxorubicin 25 mg/m^2^, bleomycin 10 U/m^2^, vinblastine 6 mg/m^2^, and dacarbazine 375 mg/m^2^ on days 1 and 15). Patients that achieved a complete response (CR) at the end of therapy did not receive IFRT, patients with PR received 20-Gy IFRT to residual nodes [16].
  Group 2 or intermediate risk (IR): patients not included in Group 1 or Group 3 were treated with 6 cycles of ABVD. Patients with a PR to therapy after the first 2 cycles received 20-Gy IFRT to residual nodal areas at the end of all chemotherapy; patients that achieved an early CR were spared all radiation.
  Group 3 or HR: patients with stages IIB, IIIB, IV A, and B were treated with 2 cycles of OEPA (prednisone 60 mg/m^2^/day on days 1–15; vincristine 1.5 mg/m^2^/day on days 1, 8, and 15; doxorubicin 40 mg/m^2^/day on days 1 and 15; and etoposide 125 mg/m^2^/day on days 2–6) and 4 cycles of COPDAC (prednisone 40 mg/m^2^/day on days 1–15; dacarbazine 250 mg/m^2^/day on days 1, 2, and 3; vincristine 1.5 mg/m^2^/day on days 1 and 8; and cyclophosphamide 500 mg/m^2^/day on days 1 and 8). Decision about dose of radiation at the end of all chemotherapy depended on response to therapy after the 2 cycles of OEPA. The initially involved areas with an early CR received 20-Gy IFRT and those with an early PR received 25 Gy.

### 2.3. Response Definitions

The response evaluation had to be performed after the fourth and second cycle of ABVD in LR and IR patients, respectively, and after the second cycle of OEPA in HR patients.

  CR was defined by the inability to palpate or identify by imaging the initially affected lymph nodes and disappearance of all symptoms. Patients with residual disease in the mediastinum were considered in CR if there was a reduction of more than 80% in the product of two dimensions of the initial mass, and the PET was negative (Deauville scores 1, 2, and 3). Patients with abdominal disease were considered in CR if the PET was negative and if the lymph node mass had decreased by more than 80% and if each residual lymph node mass did not exceed 2 cm. In addition, there should be no evidence of disease in previously affected extralymphatic areas.
  PR: A PR was defined as a reduction of tumor volume less than 80% but greater than 50% by physical examination or any imaging method, regardless of PET response, or a positive PET in a residual mass with reduction greater than 80%.
  Progressive disease (PD): histopathological evidence of disease progression in a previously healthy area or more than 50% increase in the size of known lesions.
  Relapses: biopsy-proven histopathological evidence of HL after achieving CR.

### 2.4. Statistical Methods

Times to event data were analyzed using the Kaplan–Meier [17] method and the log-rank test. OS was defined as time from registration until death from any cause. EFS was defined as time from registration until the occurrence of one of the following events: progression/relapse of disease, occurrence of a secondary malignancy, or death from any cause. The cutoff for data analysis was June 2022.

## 3. Results

**Figure 1:**
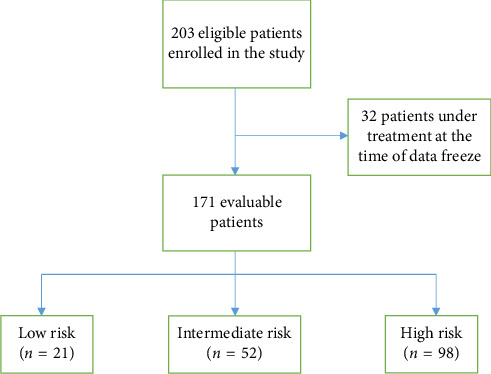
Patients enrolled in the study: between November 2012 and June 2022, 203 eligible pediatric patients were enrolled on the study. For this analysis, 171 patients were evaluable as 32 patients were still under treatment at the time of data freeze and were not included.

Between November 2012 and June 2022, 203 eligible pediatric patients were enrolled on the study. For this analysis 171 patients were evaluable as 32 patients were still under treatment at the time of data freeze and were not included (Figure 1). Median follow-up was 7.17 years (IQR: 5.28–8.71 years). In 2022, an addendum was passed to not irradiate sites of disease that achieve CR in the interim evaluation regardless of the risk group.

Of the 171 patients, 67% were male and male predominance was maintained in each risk group (LR: 67%, IR: 63%, and in HR: 67-68%). The median age was 11.9 years (range: 4–18 years). When the risk groups are analyzed, the median age was lower in the LR group of 11 years, compared with 12 years in IR and 13 years in the HR group. This difference was not statistically significant, partly due to the number of LR patients. The LR group also showed a slight increase in the incidence of mixed cellularity as opposed to IR and HR patients.

The most frequent histological subtype was nodular sclerosis in 73% of the patients. More than half of the patients had stage III (24%) and IV (29%) disease, and more than half also presented B symptoms (51%) (Table 1).

Distribution by risk groups: HR: 98 patients (57.3%), IR: 52 patients (30.4%), and LR: 21 patients (12.3%). A total of 132/171 patients (77.2%) presented with mediastinal involvement and in 74/132 (56.1%) with mediastinal bulky disease. A total of 89% of HR patients experienced B symptoms and 32.2% presented with extranodal disease (Table 2).

The response evaluation was performed by PET/CT in 147 (86%) patients. Twenty-four (14%) patients were evaluated by CT only in the first years of the guideline before PET/CT became available to the whole group.

Complete remission was achieved in the interim assessment in 95% of LR patients, 77% of IR patients, and 66% of HR patients.

Sixty-eight (40%) patients did not receive radiotherapy. One hundred and three (60%) patients received radiotherapy, of which 76% received 20 Gy and 24% received 25 Gy. Radiotherapy was omitted in 95% of the LR patients and 70% of the IR patients. Eight patients in the HR group did not receive radiotherapy because of an event (see the following).

Of the 19 patients with lung involvement, none of them relapsed and nearly 90% (17/19) did not receive radiotherapy.

The 10-year OS was 95% (95% CI: 90.7%–97.6%) for the 171 patients, 100% for LR patients, 100% for IR patients, and 93% (95% CI: 85.3%–96.4%) for HR patients.

**Figure 2:**
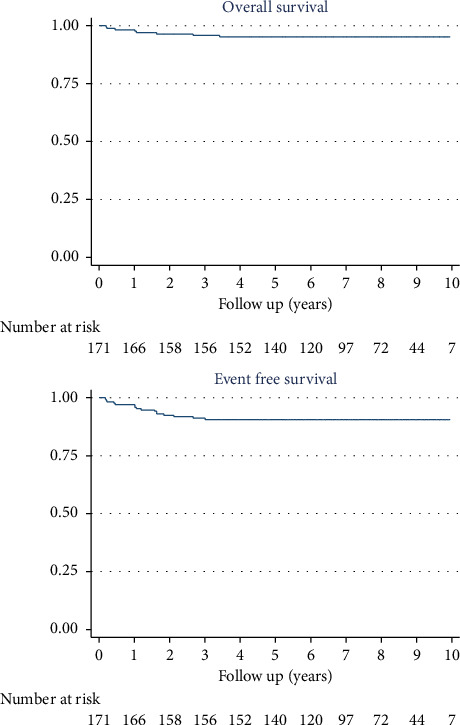
Overall survival and event-free survival of the whole cohort: the 10-year OS was 95% (95% CI: 90.7%–97.6%) and the 10-year EFS was 91% (95% CI: 85.2–94.2) for the 171 patients.

**Figure 3:**
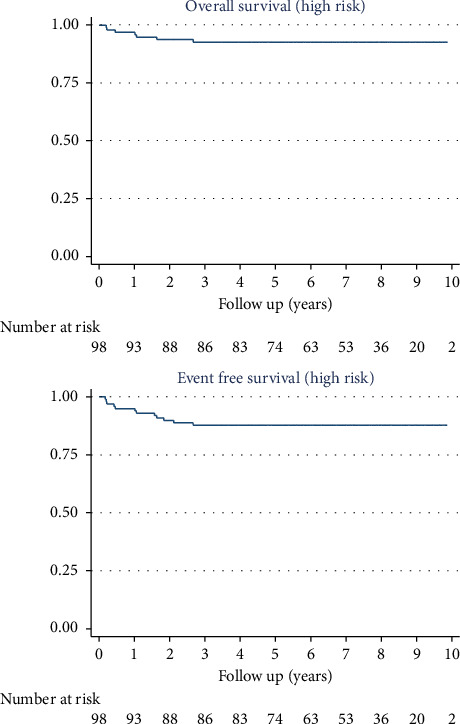
Overall survival and event-free survival of the high-risk patients: The 10-year OS and EFS for high-risk patients were 93% (95% CI: 85.3%–96.4%) and 87.8% (95% CI: 79.5–92.9), respectively.

The 10-year EFS was 91% (95% CI: 85.2–94.2) for the 171 patients, 100% for LR patients, 94.2% (95% CI: 83.2–98.1) for IR patients, and 87.8% (95% CI: 79.5–92.9) for HR patients (Figures 2 and 3).

Two patients died due to disease progression, both from the HR group (one died after a late relapse while the other patient was refractory to treatment from the beginning). Another HR patient died from secondary acute myelodysplastic leukemia immediately after the end of treatment. Two patients from the HR group abandoned treatment in CR before radiotherapy and are alive, one of them relapsed, and the other one is disease free.

We had only one loss to follow-up in a LR patient 3 years after diagnosis.

There were two early deaths in the HR group: 1 from a cause not related to treatment in complete remission and another from septic shock before his early response evaluation. Although mild to moderate myelosuppression was commonly observed in the OEPA regimen, NCI-CTC grade 3 or 4 infections were exceptional. No patient required blood transfusions. The use of stimulating factors was at the discretion of the treating physician.

Other events: 8 patients relapsed and are alive after successful salvage therapy.

Eight (8.2%) patients in the HR group did not receive radiotherapy because of an event, one suffered refractory disease, 2 abandoned therapies, 2 suffered an early death, and 3 had an early relapse. Patients with early relapses or refractory disease after OEPA–COPDAC were difficult to rescue and required anti CD30 or check point inhibitors to achieve CR and successful consolidation with autologous stem cell transplantation (ASCT) [18].

None of the 8 patients with nodular lymphocyte predominant HL relapsed.

## 4. Discussion

The collaboration presented here helped in the centralized review of patients, which translated into improvement in the standard of care, and it was essential for physicians to agree to switch to an intensive regimen leaving aside ABVD for HR patients. The decision to retain the ABVD strategy for low and IR patients had to do with the fact that the change in treatment paradigm needed to demonstrate that it was possible to use an intensive scheme such as OEPA–COPDAC in those in who needed better results and ABVD was not an option.

Carrying out a comparative analysis of this experience with another in the region is not easy because the publication rate is low in Latin America.

Regarding the publications that can be mentioned, at the time of starting this study, patients were treated without consensus between the different centers in Argentina. At that time, according to data from the Registro Oncológico Hospitalario Argentino (ROHA), survival in pediatric HL was around 85% [19]. What is more, in our previous experience until 2010 (7-PHD-96), already mentioned, the EFS was 67% in patients with advanced disease.

In 2017, a study was published which was conducted in a single institution in Argentina where all-risk patients were treated with ABVD [20]. Although they were able to omit radiotherapy at a similar percentage to our study in LR and IR patients, they also had to irradiate all HR patients. The present experience, with a median follow-up of more than 7 years and data consolidated over time, in a cohort of patients belonging to multiple centers in Argentina, with a higher percentage of patients with advanced disease, not only achieved a 10-year EFS and OS of 88% and 91%, respectively, but an intensive regimen such as OEPA–COPDAC in HR patients, after reproducing the European outcomes, allows in the current addendum, according to the evidence published by EURONET, to omit radiotherapy in HR patients that achieve CR without jeopardizing the results, something difficult to think about with ABVD as a treatment scheme. But for this, it was necessary to demonstrate that an intensive scheme like OEPA-COPDAC could be implemented in a medium-resource country.

Most of our patients were presented with advanced disease and the degree of involvement and the dimensions of voluminous disease greatly exceeded those of HIC patients with the same stage (of the 74 patients with bulky mediastinal disease, 50% presented an M/T ratio > 70%—data not shown).

The staging and early response evaluations were reviewed by the group in a periodic case presentation conference, and the objectives of reducing the number of patients who received radiotherapy as well as improving EFS in HR patients have been achieved.

We highlight that the omission of radiation in IR patients with bulky disease who achieved CR after 2 cycles of ABVD was possible in most patients without affecting EFS.

A limitation of the study is for one side, the low representation of patients with localized diseases. This has to do with the fact that the institutions that participated were reference centers where the highest risk cases were referred. On the other hand, the inclusion of NLPHL, which are treated separately from 2022.

Preliminary results were presented at ISCAYAHL in 2017 and at ASH 2022 [13, 14]. This report on a series of patients with a median follow-up of more than 7 years shows mature data that have maintained the trend observed in the group's previous reports. Our experience and the published evidence from HIC trials led us to incorporate an amendment of our treatment guideline in 2022 to not irradiate sites of disease that achieve CR in the interim evaluation regardless of risk group, as well as the adoption of the OEPA–COPDAC scheme for all risk groups.

## 5. Conclusion

The collaborative work allowed us a centralized review of the staging and response of the patients which makes this work more solid and, at the same time, to significantly improve the results of patients with advanced disease in Argentina compared with our previous experience (7-PHD-96: COPP–ABV × 6 + IFRT Bulky Disease or PR (20/25 Gy): 5yOS: 85%, 5yEFS: 67%), reduce radiotherapy doses as well as the number of patients who required radiotherapy, and finally reproduce the European experience for HR patients in a totally different context.

## Figures and Tables

**Table 1:** Patient characteristics.

Patient characteristics	
All patients (*n* = 171)	
Female	57 (33%)
Male	114 (67%)
Median age (years)	11.9 (range: 4–18)
Histology	
Nodular sclerosis	125 (73%)
Mixed celularity	35 (20%)
Lymphocyte rich	1 (0.6%)
Lymphocyte depleted	1 (0.6%)
Nodular lymphocyte predominant	8 (4.7%)
Not classifiable	1 (0.6%)
Stages	
I	16 (9.4%)
II	64 (37%)
III	41 (24%)
IV	50 (29%)
B symptoms	87 (51%)
Mediastinal involvement	132 (77%)
Bulky mediastinum	74 (56%)
Extranodal disease extension	55 (32%)
Early response evaluation	
PET/CT	147 (86%)
CT only	24 (14%)
Risk groups	
High risk	98 (57%)
Intermediate risk	52 (30%)
Low risk	21 (12%)
Radiotherapy	
No	68 (40%)
Yes	103 (60%)
20 Gy	77%
25 Gy	24%

**Table 2:** Histology and risk group.

**Histology and risk group**				
**Risk**	**Mixed celularity^∗^**		**Nodular sclerosis^∗^**	

Low (*n* = 21)	6 (29%)		13 (62%)	
Intermediate (*n* = 52)	11 (21%)		38 (73%)	
High (*n* = 98)	18 (18%)		74 (76%)	

**Risk and stage**				
**Risk**	**Stage I**	**Stage II**	**Stage III**	**Stage IV**

Low	10 (48%)	11 (52%)	—	—
Intermediate	6 (12%)	34 (65%)	12 (23%)	—
High	—	19 (19%)	29 (30%)	50 (51%)

**Extranodal involvement in high-risk patients (n = 98)**				

Lung	19 (19%)			
Liver	18 (18%)			
Bone marrow	21 (21%)			
Bone	13 (13%)			

^∗^Numbers do not add to 100% as only mixed cellularity and nodular sclerosing histology is shown, the rest are “other.”

## Data Availability

The data that support the findings of this study are available on request from the corresponding author. The data are not publicly available due to privacy or ethical restrictions.

## Nomenclature

AHOPCA: Asociación de Hemato-Oncología Pediátrica de Centro América
ABVD: Doxorubicin, bleomycin, vinblastine, and dacarbazine
ABV: Doxorubicin, bleomycin, and vinblastine
COPDAC: Cyclophosphamide, vincristine, prednisone, and dacarbazine
COPP: Cyclophosphamide, vincristine, prednisone, and procarbazine
CR: Complete response
EFS: Event-free survival
GATLA: Grupo Argentino para el tratamiento de Leucemias Agudas
GPOH: German Pediatric Oncology Hematology
HL: Hodgkin lymphoma
HIC: High income countries
HR: High risk
ISCAYAHL: International Symposium for Childhood, Adolescent, and Young Adult Hodgkin Lymphoma
IFRT: Involved field radiotherapy
IR: Intermediate risk
LMIC: Low- and middle-income countries
LR: Low risk
OEPA: Vincristine, etoposide, prednisone, and doxorubicin
OPPA: Vincristine, procarbazine, prednisone, and doxorubicin
OS: Overall survival
PD: Progressive disease
PR: Partial response
SJ: St. Jude Children's Research Hospital

## Supporting Information

Complete Description: Figure 1: Comparison of patients with high-risk HL treated with OEPA/COPDac (blue) and Stanford V (purple) in Asociación de Hemato-Oncología Pediátrica de Centro América (AHOPCA). AHOPCA introduced this strategy for pediatric patients with HR HL and markedly improved the survival in the setting of a LMIC (Permission from Dr. Castellanos and AHOPCA). The Asociación de Hemato-Oncología Pediátrica de Centro América (AHOPCA) is one of the first regional organizations formed to address the disparity in pediatric cancer care in Central America and the Caribbean. In 2009, a new treatment guideline was implemented in AHOPCA for patients with high risk (HR) HL to be treated based on the treatment group 3 (TG-3) of the GPOH-HD2002 protocol. The German study enrolled 239 advanced-stage pediatric patients with classical cHL between November 2002 and December 2005. Boys received two courses of OEPA (vincristine, etoposide, prednisone, and doxorubicin) and girls received two courses of OPPA (vincristine, procarbazine, prednisone, and doxorubicin) during induction and four further cycles of COPDAC (cyclophosphamide, vincristine, prednisone dacarbazine/procarbazine) or COPP (girls), respectively. After completion of chemotherapy, all patients received consolidative involved field radiotherapy (IFRT) of all initially involved sites of disease. The study concluded that OPPA–COPP and OEPA–COPDAC were interchangeable for patients with advanced-stage cHL and the 5-year EFS was around 90% ^21^ AHOPCA introduced this strategy for pediatric patients with HR HL and markedly improved the survival in the setting of a LMIC compared with previous experience using the Stanford V regimen (without mechlorethamine, using cyclophospfamide instead). Following this success and increased interaction between different Latin American groups fueled a regional collaboration facilitated by the International Symposia on Childhood, Adolescent, and Young Adult Hodgkin Lymphoma (ISCAYAHL), which started in 2011 and recurs every 3 years. Figure 2. In Argentina, since its creation in 1967, the Grupo Argentino para el Tratamiento de la Leucemia Aguda (GATLA) registered 1110 pediatric patients in 7 consecutive protocols for HL until 2012 and this allowed the survival of pediatric patients with Hodgkin's lymphoma to be improved over time through collaborative work. Historically, the protocols were designed to include both adult and pediatric patients. However, since 1996 pediatric patients have benefitted from strategies designed exclusively for their needs. The 7-P-HD-96 experience that enrolled patients from 1996 through 2004 included 351 pediatric patients younger than 16 years old. Patients with HR HL received COPP–ABV (doxorubicin, bleomycin, and vinblastine) × 6 + 20 Gy IFRT or 30 Gy to sites of bulky disease and partial response, leading to 5-year EFS of 67% and a 5-year OS of 85%. These results highlighted unresolved challenges and needed improvement. ^22,23^. In 2010, the EHP-10 protocol prescribed 6 cycles of ABVD + 30-Gy IFRT for HR HL patients. The recruitment of patients to this protocol was slow due to the investigators' opposition to treating patients with a strategy with a reported relapse and progression rate of 30%. Therefore, EHP-10 closed early after only 29 patients were enrolled over 3 years, of which only nine had HR disease and of which three relapsed within the first year^9^.
