# Supplementary material for: Risk-Stratified and Response-Adapted Therapy for Pediatric Hodgkin Lymphoma in Argentina: The GATLA Experience
Source: Adv Hematol. 2025 Jul 26;2025:5453729. doi: 10.1155/ah/5453729 (PMC12317811; doi:10.1155/ah/5453729)

**Supplementary Material: Figure 1 and 2:**

**Figure 1**

Comparison of patients with high risk HL treated with OEPA/COPDac (blue) and Stanford V (purple) in Asociación de Hemato-Oncología Pediátrica de Centro América (AHOPCA). AHOPCA introduced this strategy for pediatric patients with HR HL and markedly improved the survival in the setting of a LMIC (Permission from Dr. Castellanos and AHOPCA).

**
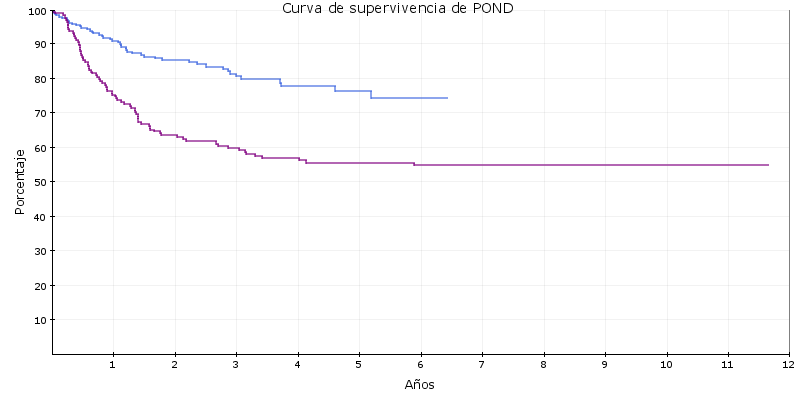
**

**Figure 2**

In Argentina, since its creation in 1967, the Grupo Argentino para el Tratamiento de la Leucemia Aguda (GATLA) registered 1110 pediatric patients in 7 consecutive protocols for HL until 2012 and this allowed the survival of pediatric patients with Hodgkin's lymphoma to be improved over time through collaborative work.


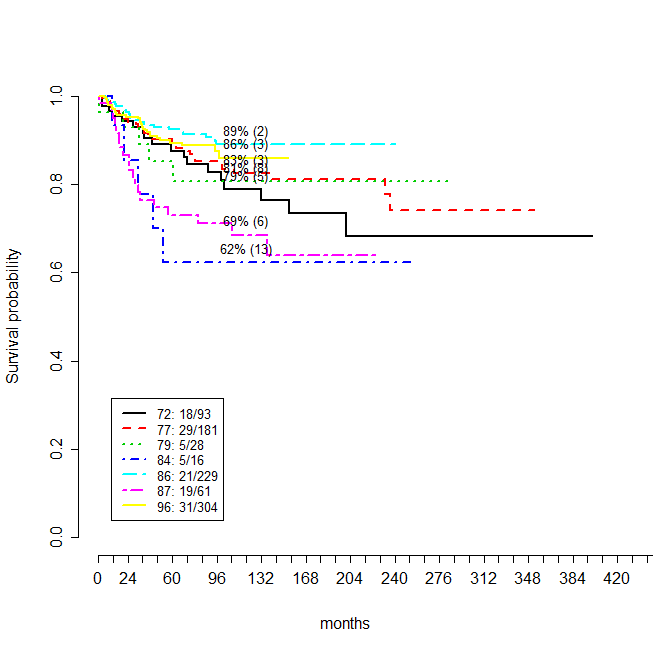

Supplement: Supporting Information — Additional supporting information can be found online in the Supporting Information section. [file 5453729.f1.docx]
